# Nanocomposite Materials: A Section of Nanomaterials

**DOI:** 10.3390/nano12020203

**Published:** 2022-01-09

**Authors:** Jordi Sort

**Affiliations:** 1 Departament de Física, Universitat Autònoma de Barcelona, 08193 Bellaterra, Spain; jordi.sort@uab.cat; Tel.: +34-935812085; 2 Institució Catalana de Recerca i Estudis Avançats (ICREA), Pg. Lluís Companys 23, 08010 Barcelona, Spain

“Nanocomposite materials” is one of the main sections of Nanomaterials and it has contributed with more than 440 publications during the last two years to increase the reputation and recognition of the journal by the scientific community.

This section focuses on publishing research in the area of multi-component or hybrid materials in which at least one of the constituent counterparts is nanometric in size [1,2,3,4,5]. Such materials take advantage of the simultaneous properties of two or more phases comprised in the composites. While the individual properties of each phase can provide, on their own, unique functionalities, very often the mutual coupling between the different types of materials offer synergetic interactions that can boost performance of the composites or even trigger new effects, not displayed by each of the constituent phases individually. In this way, nanocomposite materials are the forefront of contemporary physics, chemistry and advanced materials science technologies. The fields of application of nanocomposite materials encompass, but are not limited to, biomedicine (drug delivery, biosensors) [1], electronics and optoelectronics (sensors/actuators, recording media, MEMS/NEMS), environment, energy (supercapacitors, batteries, fuel cells) [3], automotive and aerospace (ultra-hard coatings, radiation shields) or information technologies [4] and the Internet of Things, among others.

Research interest to the section Nanocomposite Materials includes all aspects of the design, synthesis, characterization, and application of multi-phase or multi-component nanomaterials. The list of materials covered in this Section includes: protective nanocomposite coatings; self-healing nanocomposites; shape memory and shape switching nanocomposite layers; piezoelectric, magnetocaloric, magnetoelectric, or halochromic nanocomposites; metamaterials; multifunctional core-shell nanoparticles [4]; organic-inorganic hybrid composites; metal-oxide nanocomposites [5]; etc. Any aspect related to the fundamental understanding of the properties of these materials as a function of their nano-sized constituents, as well as their integration into devices is covered and will be highly recommended for publication in the “Nanocomposite Materials” Section of Nanomaterials. Our goal is to offer the readers free-to-read, high-quality papers reporting on the aforementioned cutting-edge research, aiming at further increasing the impact of the journal in the community.
